# Visual cortical responses in age-related hearing loss show evidence for compensatory neuroplasticity

**DOI:** 10.1007/s11357-025-02013-w

**Published:** 2025-11-29

**Authors:** Patricia V. Aguiar, Michelle R. Williams, Brandon T. Paul

**Affiliations:** Department of Psychology, Toronto Metropolitan University, 350 Victoria St, Toronto, ON M5B 2K3 Canada

**Keywords:** Age-related hearing loss, Neuroplasticity, Visual evoked potentials, Cross-modal, EEG, Neural oscillations

## Abstract

Sensory loss is prevalent in older adults and is associated with changes to brain structure and function. In early life, the brain compensates for sensory loss by upregulating intact senses, such as in deafness where neural sensitivity for vision increases and visual peripheral perception improves. However, it is unclear if similar neuroplastic compensation occurs in older adults with sensory loss, which would show the aging brain’s adaptability and inform sensory rehabilitation strategies. We tested for evidence of compensatory visual neuroplasticity in adults (*N* = 66) aged 53 to 80 with typical hearing or hearing loss, and if this neuroplasticity differed for visual stimuli that were or were not relevant to speech perception. Participants viewed speech-like or non-speech stimuli as we recorded cortical activity with the 64-channel electroencephalogram (EEG). Participants with more hearing loss tended to have longer cortical P1 and N1 latencies in the visual evoked potential. However, the later cortical P2 response latency decreased with more hearing loss in agreement with compensatory plasticity. Effects were independent of numerical age. Latency effects in hearing loss were more pronounced for the speech-like stimulus compared to the non-speech stimulus, but P2 responses for the non-speech stimulus showed greater cross-modal recruitment of the temporal cortex. Findings show for the first time that compensatory plasticity operates on later cortical P2 responses in older adults, is not explained by numerical age, and differs for speech and non-speech events. However, P1 and N1 responses in networks coding for visual speech may be sensitive to sensory decline.

**Supplementary Information:**

The online version contains supplementary material available at https://doi.org/10.1007/s11357-025-02013-w.

## Introduction

Gradual decline of senses such as hearing, vision, and touch is common in aging [19] which can influence behavior, cognition, and neural integrity [33, 35, 57, 58]. Some senses are more likely to decline than others [16, 20, 26]. For instance, age-related hearing loss affects one third of adults above the age of 60 and more than half of those above 70 [45]. Age-related hearing loss has many causes, such as noise injury, ototoxic exposure, and trauma accumulated across the life span, in addition to cochlear biological aging and other genetic factors [34]. It can impair speech perception and spatial listening, especially in noisy environments, and is the leading cause of years lived with disability in older adults worldwide (GBD 2019 Ageing Collaborators, 2022). Like aging, hearing loss is correlated with cognitive decline [44] and low psychosocial well-being [10]. Managing these outcomes requires investigation into the neural changes that follow both aging and sensory loss.

Studies on hearing loss and deafness that occur early in life show that healthier senses such as vision or touch upregulate to compensate for declines in hearing, demonstrating the remarkable flexibility of the nervous system to adapt to its sensory experience. In behavioral research, for example, individuals with deafness show superior performance on visual tasks such as motion detection [69] and visual localization [18] compared to controls with typical hearing. In neuroimaging, individuals with deafness have larger neural responses to visual or tactile stimulation [25, 42], suggesting enhanced responsivity within these senses. Similarly, improvements in auditory function are found in cases of blindness [39, 65, 73]. These perceptual and neural enhancements following sensory loss are causally linked to neural plasticity [47]. A *cross-modal* form of neuroplasticity occurs when neurons shift their response preference from one sense to another, such as when neurons of the deaf auditory cortex become more responsive to visual input [25, 37]. *Intra-modal* plasticity occurs when sensory processing improves within its respective cortex, demonstrated by stronger visual cortex responses to movement in individuals with early deafness [7]. Cross- and intra-modal plasticity are considered compensatory processes as they are presumed to offset deficits caused by unisensory loss. Recent proposals suggest that compensatory plasticity in deafness and hearing loss unfolds in higher-order areas of the sensory cortex and arises through pre-existing top-down connections of healthy senses that upregulate based on the organism’s experiences [38].

Whether compensatory plasticity occurs in aging is unclear. Unlike total deafness or blindness that occurs at birth or in early life, milder forms of sensory decline in later life begin after the brain has matured with an established set of sensory experiences. Few studies have investigated compensatory plasticity after partial hearing loss in adults, some of which extend into older adult populations. Most use electroencephalography (EEG) to measure cortical responses to visual stimuli such as motion or images of objects. Compared to control subjects with normal hearing, adults with hearing loss appear to produce visual evoked potentials (VEPs), including P1, N2, and P2 components, with shorter latencies and sometimes larger amplitudes [13, 14, 30, 43]. Shorter VEP latencies are often taken as evidence of compensatory visual plasticity, since they likely signal a strengthening of visual processing. Current research, however, does not agree on which evoked potentials exhibit these effects (e.g., N1 vs. P2, or latencies vs. amplitudes) nor where they occur across scalp regions (e.g., occipital vs. temporal). It is also unclear if cross- or intra-modal plasticity accounts for these effects. One study used source analysis on EEG responses and found descriptively higher visual cross-modal activation of auditory cortex in a participant group with hearing loss, but this did not significantly differ from controls without hearing loss [71]. Cross-modal plasticity is, however, reliably detected in animal models of partial noise-induced hearing loss [50, 67]

While VEP latencies are shown to decrease with hearing loss, the aging and EEG literature routinely shows that VEP latencies increase with age [11, 59, 68, 70, 72]. These findings are attributed to the decline of the central nervous system and not due to peripheral causes, because participants had normal- or corrected-to-normal visual acuity. Alain et al.[2] measured evoked potentials for visual, auditory, and somatosensory stimuli in older and younger adults. Latency increases were found for all stimulus types and were correlated within participants. These outcomes were explained as the result of global age-related degeneration (i.e., a “common cause”), although there is debate over whether age-related sensory decline has a common cause or if each sense declines along its own trajectory [5, 17]. Nonetheless, age-related cortical decline and compensatory sensory plasticity predict opposite effects on VEPs: shorter latencies in hearing loss reflect visual neural compensation, while longer latencies reflect age-related neural decline. To our knowledge, no current study has tested for these contrasting effects in the same individuals.

Here we used EEG to measure VEPs in middle-aged and older adults with varying degrees of hearing loss. For the P1, N1, and P2 VEP, we predicted earlier latencies and larger amplitudes with more hearing loss, consistent with expectations for visual compensatory plasticity. In addition, we tested whether the stimulus content matters for suspected visual plasticity. Research on cross-modal plasticity in cochlear implant users suggests different compensatory mechanisms are employed for visual speech stimuli compared to non-speech stimuli [29], which may be due to differences in non-speech and speech-specific neural pathways [9]. Therefore, we included non-speech and speech-like stimuli in our design. Tests of these hypotheses were complemented by exploratory source analysis to analyze cross- and intra-modal contributions. We also performed exploratory analyses on the relationship between hearing acuity and neural oscillatory activity. To our knowledge no prior work has examined visual event-related power fluctuations or inter-trial phase coherence as a function of hearing acuity in older adults, which adds novelty to this study.

## Methods

### Participants

We recruited 76 middle-aged and older adults to participate in our study. Ten participants had technical failures or excessive noise during EEG recording and were omitted from analysis. The remaining 66 participants were aged 53 to 80 years (*M* = 69.98, SD = 5.51). Thirty-six individuals self-reported as women, 29 as men, and 1 as gender-fluid. Fifty-eight participants identified as White, 3 as Black, 2 as East/Southeast Asian, 1 as East Asian, 1 as Middle Eastern, and 1 as South Asian. Participants were recruited from databases maintained at Toronto Metropolitan University. All participants provided written informed consent, and the ethics protocol was approved by the Research Ethics Board at Toronto Metropolitan University (REB #2021–415). Participants received $20 CAD per hour for their time.

**Fig. 1:**
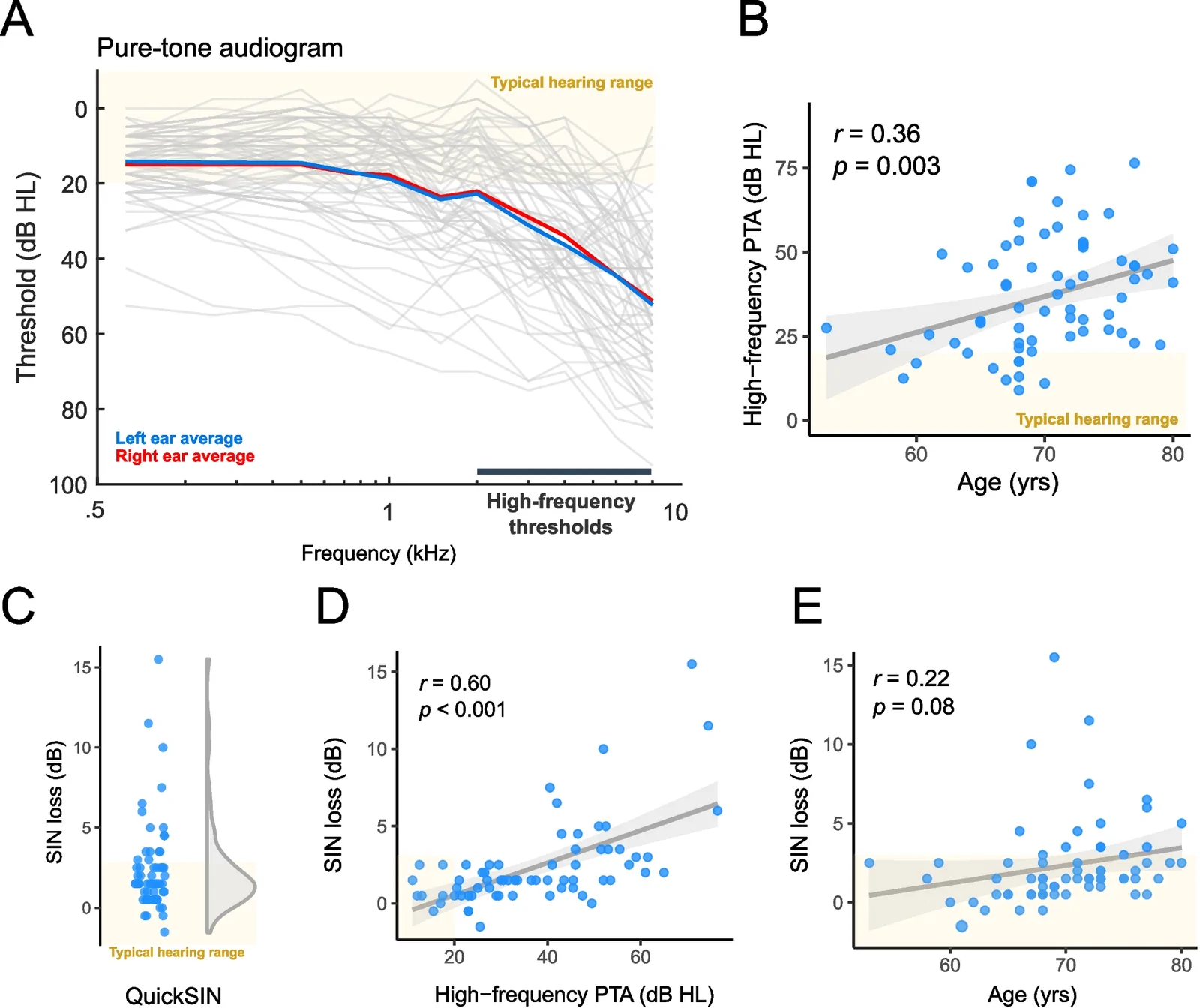
Hearing ability, age, and their relationship. **A** The audiogram, showing hearing thresholds in decibels hearing level (dB HL) as a function of pure-tone sound frequency. Higher thresholds (worse hearing) are conventionally plotted on an inverted ordinate. Thick red and blue lines respectively represent the average right and left ears. Individual audiograms, averaged across ears, are plotted as thin gray lines. The high-frequency range from 2 to 8 kHz is distinguished by the thick dark line, and the typical hearing range is highlighted in yellow. **B** High-frequency pure tone average (PTA) as a function of age. **C** Distribution of scores for QuickSIN, a speech-in-noise measure. Raw jittered data points are shown beside a density estimate for the distribution. Higher values indicate worse speech-in-noise perception. **D** Scatter plot of high-frequency PTA and QuickSIN and **E** age and QuickSIN. For (**B**), (**D**), and (**E**), individual data points are participants. Least squares lines are shown in gray, bounded by 95% confidence intervals

All participants underwent pure-tone audiometry (Fig. 1A), the gold standard test of hearing loss that estimates 50% detection thresholds for sinusoidal (pure) tones measured in decibels hearing level (dB HL). Higher thresholds indicate worse hearing acuity. There is no international consensus on the degree of threshold shift at which hearing loss begins, being that it is a continuous measure. However, individuals are commonly graded as having hearing loss when thresholds exceed 20 dB HL in the better ear, when averaged from 500 Hz to 4 kHz [52]. Approximately half of the participants had hearing loss according to this classification, including 30 with mild loss > 20 dB HL, and 3 with “moderate” loss > 40 dB HL. The remainder had no measurable hearing loss. However, threshold shifts in aging most often occur above 2 kHz and are most extreme at 6 and 8 kHz. Consistent with past public health studies on age-related hearing loss [64] and in cross-modal plasticity [30], our measure of hearing acuity was an average of “high-frequency” (2–8 kHz) thresholds, that was also averaged across both ears. Consistent with convention, we refer to this measurement as the high-frequency pure-tone average (PTA). Hearing acuity tends to worsen with age (Fig. 1B). No participant had a history of hearing aid use.

### Stimuli and materials

**Fig. 2:**
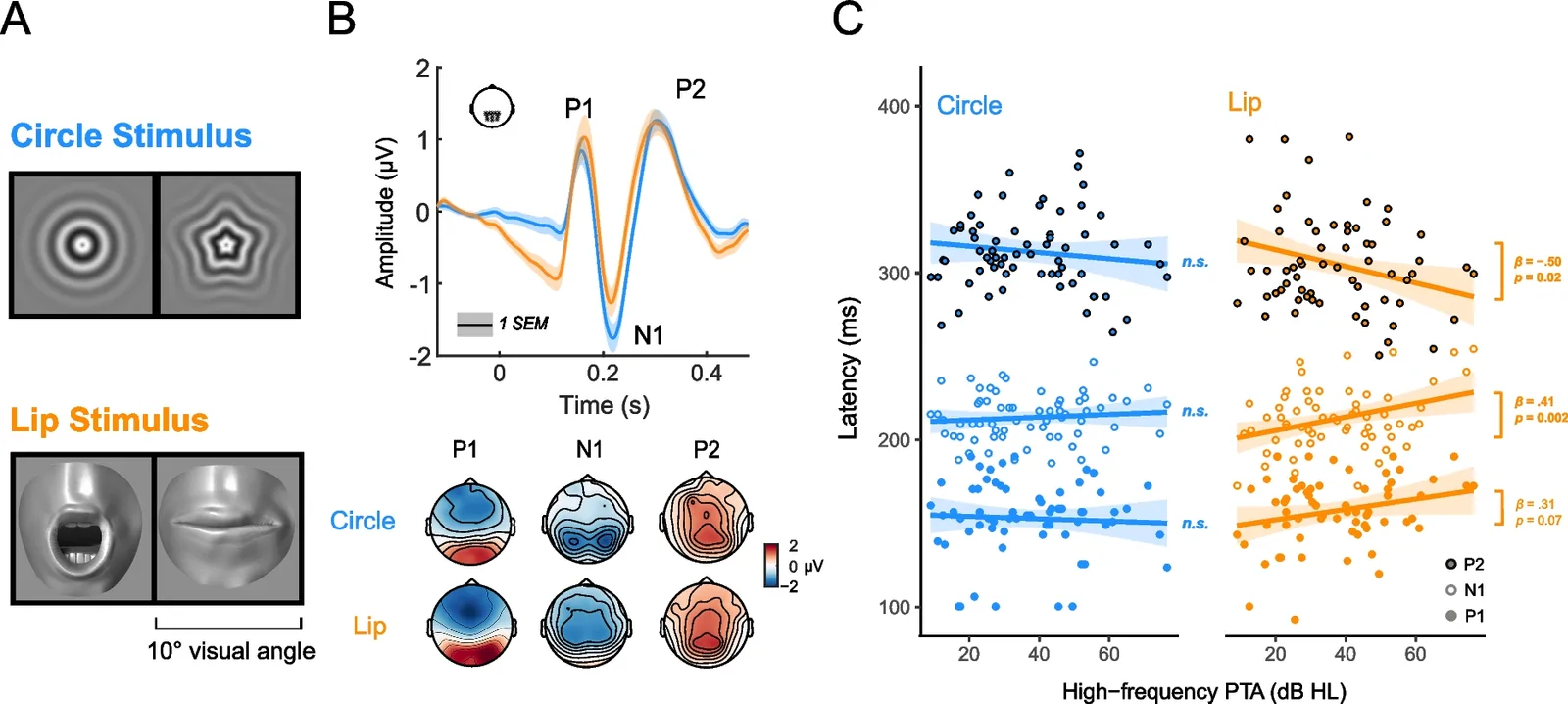
Visual evoked potentials (VEPs) and their relationship to hearing thresholds. **A** Stimuli used to evoke cortical potentials. **B** VEPs for the circle and lip stimuli. Shaded regions are 1 standard error of the mean. Topographies for the N1 and P2 components are shown for each stimulus underneath the waveform, and the color bar reflects the voltage distribution. **C** Scatterplots showing VEP latencies for each component as a function of high-frequency PTA. Individual dots are raw data from single subjects, with solid circles reflecting P1 responses, open circles reflecting N1 responses, and dark-bordered circles reflecting P2 responses. The solid lines are the predictions from the mixed model, and shaded confidence bounds are the 95% confidence intervals. Coefficients from simple slopes analysis and associated p-values for significant effects are shown to the right of each VEP; n.s., not significant

Participants were seated in a sound-attenuated and electrically shielded room. They viewed two types of stimuli that evoked visual cortical potentials. The first stimulus (Fig. 2A, top row) has been widely used in research on cross-modal plasticity and deafness or hearing loss [13, 14, 22, 23, 30] and consists of two alternating frames of high-contrast concentric gratings. The first frame consists of a sinusoidal concentric grating (0.8 c/deg) resembling a circle. The second frame is a radially modulated version of the concentric circles, consisting of 5 bumps resembling a star. When these frames alternate, individuals perceive apparent motion [23]. We refer to this as the “Circle” stimulus.

The second stimulus, herein the “Lip” stimulus, approximated visual speech (Fig. 2A, bottom row). The Lip stimulus consisted of two alternating frames taken from 3D models of the Preston Blair phoneme series [49] used previously in research on automated text-to-speech animation [32, 51] and is designed to convey audiovisual speech. Each frame was greyscaled to match the background of the Circle stimulus. One frame was an image of a closed mouth, and the second frame was a mouth shaped to articulate “Oh” (/oω/). Like the Circle stimulus, the alternating frames could be perceived as apparent motion of a mouth. The stimulus may be perceived as a viseme (visual analogue of a phoneme), where closed-mouth consonants transition to the vowel of the second frame such as “Boh”, “Moh,” or “Poh.”

Participants were seated and distanced from a computer monitor so that each image frame subtended 10 degrees of visual angle. For both stimulus types, each frame was presented for 600 ms and alternated continuously until the end of the block. A total of 500 alternations were presented for each stimulus, totaling 1000 events. Three participants did not have EEG recorded while viewing the Lip stimulus. Therefore, there are 64 participants included in analyses involving the Lip stimulus.

### Procedure

Before the study, participants provided informed consent and demographic information in an online form. After arriving on-site, participants first underwent pure-tone audiometry. We measured thresholds for pure tones spaced by half-octave steps from 125 Hz to 8 kHz in each ear using the Hughson–Westlake procedure, with pulsed pure tones [41]. In addition, we tested speech-in-noise ability using the QuickSIN test [36]. QuickSIN consists of a female talker of American English reading the Harvard *IEEE* Sentence list and is calibrated to 65 dB sound pressure level (SPL). Sentences are presented in 4-talker babble noise. Participants listen to 12 lists of sentences and repeat them back to the experimenter. Each list has 6 sentences that decrease in signal-to-noise ratio. Performance is scored as the sum of words correctly perceived in each list, which is converted into speech-in-noise (SIN) “loss”, by subtracting 25.5 [36]. Values below 3 dB are considered normal performance. The final speech-in-noise loss was taken as the median performance across the twelve lists. We note that one participant had produced an outlier QuickSIN performance of 25 dB SIN loss, more than 8 standard deviations away from the mean, and they were removed from the analysis.

Afterwards, we tested participants’ visual acuity using the Freiburg visual acuity test (FrACT) [4]. Participants were able to use corrective lenses (eyeglasses, contacts). FrACT performance is expressed as logarithm of the minimum angle of resolution (logMAR) with values below 0.3 representing typical vision (20/40 vision). One participant was measured at 0.35 logMAR, but their exclusion did not affect statistical outcomes, and they were retained in the analysis.

Following sensory testing, participants were fitted with an electrode cap for EEG recording while they viewed short blocks of circle and lip stimuli. Each block consisted of 100 trials, where each trial was the onset of a frame of each stimulus (e.g., 50 trials for frame 1 and 50 trials for frame 2). Blocks did not alternate between stimuli; all 500 presentations for one stimulus occurred before the next stimulus type was presented. The order of stimuli was randomly determined for each person. At the start of each block, a black fixation cross appeared for 2.5 s before stimuli presentation began. During the task, participants were instructed to sit as still as possible and focus their eyes on the center of the image. At the end of each block, participants were encouraged to take a short break. They initiated the next block by keypress when they were ready. After recording, participants were able to clean conductive gel from their hair and were paid for their time.

### EEG recording and preprocessing

The EEG was recorded at 2048 Hz using a BioSemi Active2 amplifier across a 64-channel montage aligned with the international 10–20 system. Left and right mastoids were also recorded for offline re-referencing. All offline EEG processing was conducted in MATLAB R2021a using the Fieldtrip toolbox [54]. First, we used *ERPlab* to shift triggers by 20 ms to account for the delay between event code and the onset of the stimulus on the LCD monitor used for stimulus presentation, rounding to the nearest value [48]. Data were then re-referenced to the linked mastoids, epoched from − 100 to 500 ms relative to stimulus onset at 0 ms and were downsampled to 512 Hz. Trials with amplitudes exceeding ± 150 µV were considered artifactual and were removed. Afterward, EEG data were decomposed using Independent Components Analysis (ICA). Components for eye blinks, horizontal eye movements, and other myogenic artifacts were removed. Two or fewer components were removed per participant. Following ICA, noisy channels were replaced with estimates derived from neighboring channels using spline interpolation.

### Visual evoked potentials

All trials were filtered between 1 and 30 Hz using a 4th order Butterworth filter, and were baseline corrected to the − 100 to 0-ms interval before stimulus onset (i.e., a frame change). Trials were then averaged to obtain the VEP for each stimulus type. We expected evoked potentials to reach an amplitude maximum across parietal and occipital sensors, and we averaged the evoked response across 11 channels in this region (P1, Pz, P2, P4, PO3, POz, PO4, O1, Oz, O2). The P1 response had occipital activation and channel Iz (the inion) was included in the sensor average for this component. The latency for each VEP component was calculated as the time point corresponding to half the area under the curve (i.e., the 50% area latency), and the amplitude was an average of the voltages ± 10 ms around the 50% area latency. These metrics were chosen over peak amplitudes and latencies because they show greater robustness to noise [74].

### Time–frequency analysis

Using the Fieldtrip toolbox, we applied the multi-taper convolution method with Hanning tapers to estimate time–frequency representations of oscillatory power for single trials. Estimation involved sliding time windows in 50-ms steps across a frequency band of 5–30 Hz with 1-Hz resolution. The length of single trials was zero-padded to the next power of two for smoother estimation. Lower frequencies could not be estimated due to the short length of the trial. Time–frequency magnitudes were then averaged across all trials as a calculation of total oscillatory power. Power values across the entire trial were normalized by calculating the decibel change from the pre-stimulus baseline [10*Log_10_ (post-stimulus/pre-stimulus)], where the baseline was the period from − 1 to 0 s relative to stimulus onset set at 0 s.

### Inter-trial phase coherence

In the Fieldtrip toolbox, Morlet wavelets were used to decompose single trials into their complex Fourier spectrum from 5 to 30 Hz (1-Hz resolution) across each epoch in 10 ms steps. The length of single trials was zero-padded to the next power of two. The Fourier spectrum was divided by its amplitude, and to calculate inter-trial phase coherence, phase angles were summed and normalized by the number of trials. Phase coherence scales from 0 to 1, with 1 indicating perfect coherence across trials.

### Source analysis

Minimum norm estimation (MNE) was used to compute cortical activation of VEPs in source space. The default Biosemi 64-channel templates for the surface position of electrodes were realigned to the Montreal Neurological Institute (MNI) template in the Fieldtrip toolbox to create volume conduction models using the boundary element method (BEM). Note that we did not have digitized sensor positions for each person, and so the default template was applied to all participants. Single trial data were re-referenced to the common average and filtered from 1 to 30 Hz. For MNE analysis, data were pre-whitened, and the lambda coefficient for regularization was set to 3. MNE-derived source activations across the entire epoch were calculated across all voxels. We used the MNI atlas to pick regions of interest (ROIs) for testing cross- versus intra-modal assumptions based on the observed distribution of activation across voxels.

Based on grand average inspection, cortical activations were extracted from two ROIs: right middle temporal gyrus (auditory activation) and right inferior occipital gyrus (visual cortical activation). Each time series of source activation was baseline corrected by *z*-score [(post − mean(pre))/std(pre)]. Due to the use of standard templates instead of individualized head models, activation strength in ROIs across participants varied widely, but was stable within subjects (*p*’s < 0.002). Consequently, we computed a normalized index of cross-modal activation by subtracting the visual activation from the auditory activation and then dividing by the sum of the two ROIs [(AUD − VIS)/(AUD + VIS)]. Values close to − 1 reflect more visual activation compared to auditory activation, and values close to + 1 reflect more auditory activation compared to visual activation. Note that the index can exceed ± 1 because the baseline correction by z-scoring could lead to negative values.

### Statistical analysis

In R [63], we used linear mixed-effects models from the *lme4* package [6] to predict cortical responses from participant hearing thresholds across both stimulus types. Stimulus type was a categorical within-subjects variable with two levels (Circle and Lip) and deviation coding (− 0.5 = Circle, + 0.5 = Lip). The Circle stimulus was the reference level. Age in years was included as a covariate. Models also included by-participant random intercepts and random slopes across the two stimulus types. The model formula was:

*cortical response* ~ hearing threshold * stimulus type + *age* + (1 + *stimulus type* | *participant ID*).

Outcome variables representing the *cortical response* included VEP amplitudes and VEP latencies. Models with P2 latencies did not include the random slope for stimulus because the models would not converge. For some models, random slopes and intercepts were left uncorrelated to facilitate convergence. Exploratory analysis was conducted on oscillatory power, inter-trial phase coherence, and on the cross-modal index for source activations. Analysis of Variance (ANOVA) with degrees of freedom adjusted by the Satterthwaite method assessed the statistical significance of model terms. Simple slopes were used to interpret interactions between hearing thresholds and stimulus type. No evidence of misspecification or over/underdispersion was found after model residuals and other diagnostics (e.g., QQ plots) were inspected using the *DHARMa* package in R. Given the association between high-frequency hearing thresholds and participant age (Fig. 1), we calculated variance inflation factors (VIFs) for mixed effects models. VIFs were below 1.5 for all continuous variables, suggesting no problematic linear dependence between age and hearing thresholds. Pearson correlation was used to measure linear relationships between behavioral variables. The alpha criterion for type 1 error was set at 0.05.

## Results

### Hearing performance

Participant hearing behaviors are plotted in Fig. 1. Audiograms plotted in Fig. 1A show that thresholds, on average, were within normal limits below 1 kHz and then elevated in the high-frequency range of 2–8 kHz. This pattern is consistent with high-frequency sloping thresholds typical of age-related hearing loss. Hearing thresholds averaged across these frequencies (high-frequency PTA) ranged from 9 to 76.5 dB HL (*M* = 36.8, SD = 16.45) and are plotted as a function of age in Fig. 1B. Although age and high-frequency PTA were significantly correlated, this relationship was weak (*r* = 0.36, *p* = 0.003), sharing only ~ 13% of their variance. QuickSIN performance, expressed as speech-in-noise (SIN) loss in decibels (Fig. 1C), ranged from − 1.5 dB to 15.5 dB (*M* = 2.3, SD = 2.8), where < 3 dB represents “normal” hearing or no loss. Fourteen participants had SIN loss > 3 dB. High-frequency PTA was positively correlated with SIN loss (*r* = 0.60, *p* < 0.001; Fig. 1D), suggesting those with worse hearing acuity have worse speech-in-noise performance. Age was not correlated with SIN loss (*p* = 0.08; Fig. 1E). Results suggest that participants varied in hearing ability and age with many showing evidence of age-related hearing loss.

### Visual evoked potential latency effects

Figure 2B shows grand average VEPs with clear P1, N1, and P2 for both Lip and Circle stimuli. The P1 amplitude reached a maximum in occipital sensors, and the N1 and P2 reached amplitude maxima in occipital and parietal sensors. An early negative-going potential appears in the grand average before the P1, only for the Lip stimulus. Inspection of individual VEPs indicated that this early negativity was not present in all participants and was not identifiable for the Circle stimulus. Therefore, it was not analyzed further.

We hypothesized shorter VEP latencies with worse hearing acuity due to suspected visual compensatory plasticity. Modeled effects of high-frequency PTA on VEP latencies for each component are shown in Fig. 2C. A mixed model for P1 latency (Table 1) found no main effects of high-frequency PTA (*p* = 0.39), age (*p* = 0.28), or stimulus type (*p* = 0.22). There was an interaction between stimulus type and PTA (*p* = 0.049), but the simple effect of PTA on P1 latency was not significant for the Lip (*p* = 0.07) or Circle stimulus (*p* = 0.67). For the N1 latency (Table 2), there was a main effect of high-frequency PTA (*p* = 0.023) and an interaction between high-frequency PTA and stimulus type was significant (*p* = 0.029). Simple slopes analysis showed delayed N1 latencies with worse hearing for the Lip stimulus (*β* = 0.41, SE = 0.13, *p* = 0.002) but not the Circle stimulus (*β* = 0.08, SE = 0.13, *p* = 0.51). Age was not significant (*p* = 0.88), but an effect of stimulus type (*p* = 0.034), suggested Lip N1s were ~ 13 ms earlier than Circle N1s. Overall, the association between hearing thresholds and P1 and N1 latency opposed predictions of compensatory visual plasticity. However, consistent with our hypotheses, these effects depended on the speech likeness of the stimulus. N1 latencies increased an estimated 4.1 ms per 10 dB elevation in hearing thresholds in the Lip but not Circle stimuli. A similar ~ 3.2-ms effect was found for the P1 latency, but this was not significant.

**Table 1:** P1 latency

Predictor	Slope	S.E	*F*-statistic	D.F	*p*-value
High-freq. PTA	0.1198	0.1407	0.7250	1, 63.024	0.39772
Condition	− 9.6285	7.7023	1.5627	1, 63.486	0.21586
Age	0.4511	0.4169	1.1709	1, 61.400	0.28344
High-freq. PTA*condition	0.3804	0.1891	4.0475	1, 63.003	**0.04852**

*PTA* pure tone average, *S.E.* standard error, *D.F.* degrees of freedom

**Table 2:** N1 latency

Predictor	Slope	S.E	*F*-statistic	D.F	*p*-value
High-freq. PTA	0.24446	0.105	5.3973	1, 62.998	**0.02341**
Condition	− 12.86842	5.92	4.7262	1, 62.088	**0.03353**
Age	− 0.04685	0.30324	0.0239	1, 61.286	0.87773
High-freq. PTA*condition	0.32443	0.14517	4.9947	1, 61.777	**0.02905**

*PTA* pure tone average, *S.E.* standard error, *D.F.* degrees of freedom

For P2 latency (Table 3), there were main effects of hearing thresholds (*p* = 0.039) and age (*p* = 0.005) but no main effect or interactions with stimulus type (*p*’s > 0.18). Age and hearing thresholds had diverging effects on P2 latencies. The model estimated a 3.4 ms reduction in latency per 10 dB elevation in hearing thresholds (Fig. 2C), but a 1.4 ms increase in latency for each year in age. Earlier P2 latencies with worse hearing acuity are consistent with predictions of compensatory visual plasticity, while delayed latencies are consistent with aging effects [2].

**Table 3:** P2 latency

Predictor	Slope	S.E	*F*-statistic	D.F	*p*-value
High-freq. PTA	− 0.3428	0.1627	4.4404	1, 60.845	**0.039227**
Condition	4.0991	9.2842	0.1949	1, 61.500	0.660393
Age	1.3927	0.4817	8.3575	1, 59.182	**0.005363**
High-freq. PTA*condition	− 0.3105	0.2280	1.8553	1, 61.001	0.178180

*PTA* pure tone average, *S.E.* standard error, *D.F.* degrees of freedom

### Visual evoked potential amplitude effects

No main effects or interactions were found for P1 amplitudes (*p*’s > 0.40; Table 4) and no main effect for N1 amplitude was found (*p*’s > 0.15; Table 5). The interaction between hearing thresholds and stimulus type was significant for N1 amplitude (*p* = 0.047), but simple slopes analysis did not find effects of hearing thresholds for the Lip (*β* = 0.02, *p* = 0.09) or Circle stimulus (*β* =  − 0.01, *p* = 0.60). For P2 amplitude (Table 6), only a main effect of hearing thresholds was found (*p* = 0.0002) suggesting a ~ 0.3 µV decrease in P2 amplitude for each 10 dB increase in hearing thresholds. Therefore, there were minimal effects on VEP amplitudes, apart from a reduction in P2 with worse hearing acuity.

**Table 4:** P1 amplitude

Predictor	Slope	S.E	*F*-statistic	D.F	*p*-value
High-freq. PTA	− 0.0001962	0.0102590	0.0004	1, 62.594	0.9848
Condition	− 0.1545220	0.4861289	0.1010	1, 62.422	0.7517
Age	0.0167233	0.0304499	0.3016	1, 61.139	0.5849
High-freq. PTA*condition	0.0100813	0.0119286	0.7142	1, 61.999	0.4013

*PTA* pure tone average, *S.E.* standard error, *D.F.* degrees of freedom

**Table 5:** N1 amplitude

Predictor	Slope	S.E	*F*-statistic	D.F	*p*-value
High-freq. PTA	0.007833	0.009311	0.7077	1, 64.524	0.40331
Condition	0.723609	0.498478	2.1073	1, 63.186	0.15155
Age	0.038782	0.027057	2.0546	1, 62.677	0.15672
High-freq. PTA*condition	0.024739	0.012225	4.0952	1, 62.903	**0.04726**

*PTA* pure tone average, *S.E.* standard error, *D.F.* degrees of freedom

**Table 6:** P2 amplitude

Predictor	Slope	S.E	*F*-statistic	D.F	*p*-value
High-freq. PTA	− 0.029493	0.007450	15.6732	1, 61.231	**0.0001987**
Condition	0.573451	0.358124	2.5640	1, 61.122	0.1144748
Age	− 0.009731	0.022108	0.1937	1, 59.759	0.6614094
High-freq. PTA*condition	− 0.011417	0.008788	1.6879	1, 60.693	0.1987899

*PTA* pure tone average, *S.E.* standard error, *D.F.* degrees of freedom

To summarize, most effects of hearing acuity on VEPs opposed our hypotheses. P2 (but not P1 or N1) amplitudes were smaller with worse hearing. P1 and N1 latencies decreased with worse hearing acuity, but only significantly so for the N1. However, consistent with compensatory visual plasticity, P2 latencies decreased with worse hearing acuity. Since the effect of age on the P2 latency was in the opposite direction, the visual P2 may change along separate trajectories for aging and hearing loss. Overall effects were more pronounced for the Lip stimulus, but this was only statistically detected for N1 and P1 latencies. To facilitate a visual comparison of these effects, see Supplemental Fig. 1 which plots VEP waveforms by groups of individuals with worse hearing sensitivity (> 35 dB HL) and those with comparatively better hearing (< 35 dB HL).

### Time–frequency analysis of visual cortical responses

**Fig. 3:**
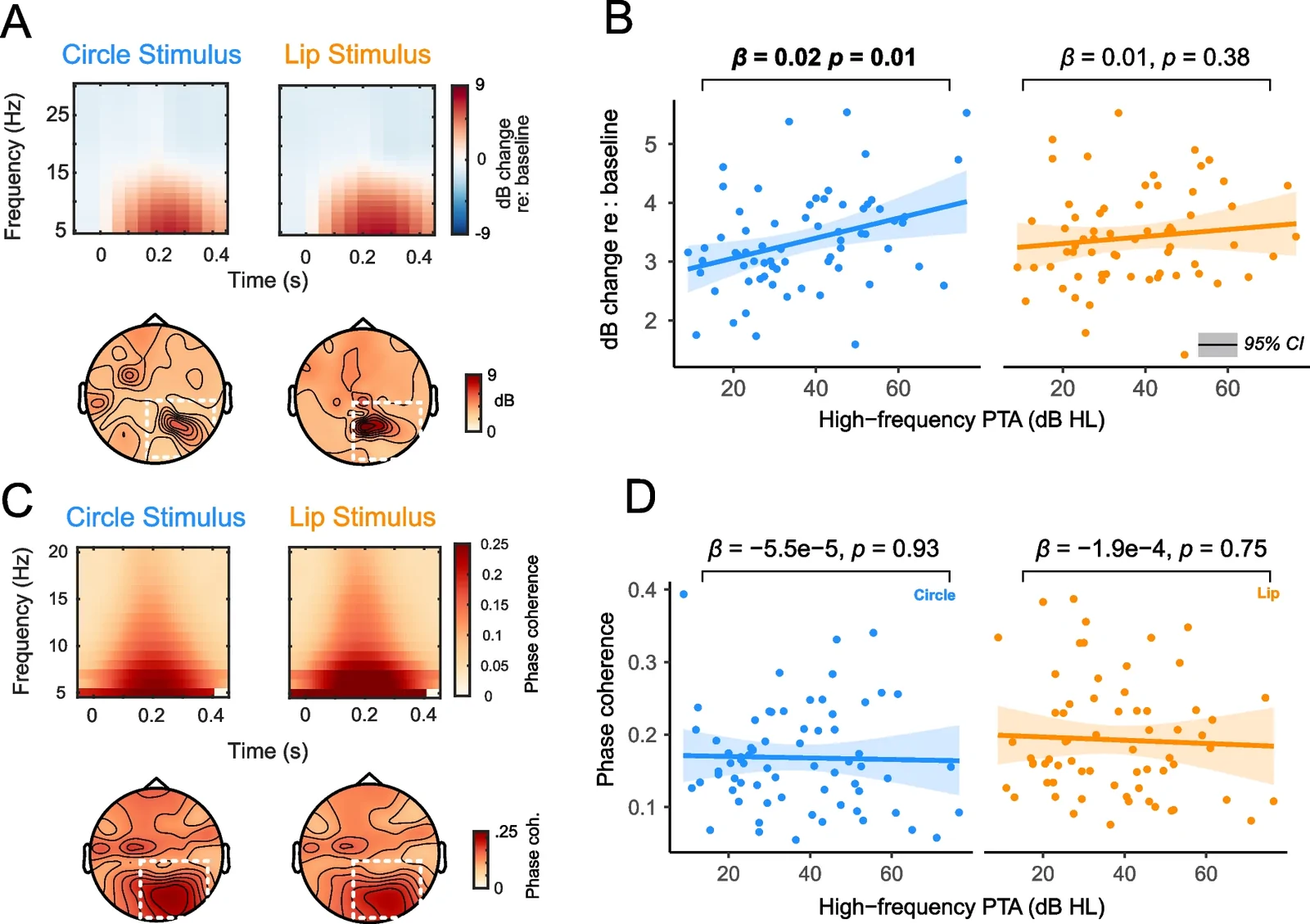
Neural oscillations elicited by each stimulus and their relationship to hearing thresholds. **A** Time–frequency plots showing modulations of total power after the onset of each stimulus type. Topographies for the power increase are shown for each stimulus underneath the spectral plots, and the color bar reflects the increase in power as a decibel relative to the − 0.1 to 0 s baseline. The white dashed box on each topography contains the sensors used in statistical analysis. **B** Scatterplots showing 5–15 Hz power as a function of high-frequency PTA. **C** Time–frequency plots showing inter-trial phase coherence calculated across all trials. Topographies for the phase coherence values are plotted underneath spectral plots; outlined regions were sensors used in the analysis. **D** Scatterplots showing 5–15 Hz phase coherence as a function of high-frequency PTA. For both (**B**) and (**D**) individual dots are single subjects, the dotted or solid lines are the predictions from the mixed model, and shaded confidence bounds are the 95% confidence intervals. Coefficients for simple slopes analysis and associated *p*-values are shown above each scatter plot

Time–frequency analysis representing total oscillatory activity during the trial epoch is shown for Circle and Lip stimuli in Fig. 3A. Both stimuli elicited an increase in power in right lateralized occipital sensors from 0.2 to 0.4 s after stimulus onset and from 5 to 15 Hz. A mixed effects model predicting total power averaged across these dimensions in Table 7 found a main effect of stimulus type (*p* = 0.011), and an interaction between stimulus type and high-frequency PTA (*p* = 0.015). Shown in Fig. 3B, the coefficient between power and PTA for the Circle stimulus was statistically significant (*β* = 0.02, SE = 0.1, *p* = 0.01). This association was not significant for the Lip stimulus (*β* = 0.01, SE = 0.1, *p* = 0.36). This result suggests a 0.2 dB increase in oscillatory power in response to the Circle stimulus per 10 dB elevation in high-frequency PTA. No effect was present for the Lip stimulus. Age (*p* = 0.52) and the main effect of high-frequency PTA (*p* = 0.07) were not significant in this model. We note that one participant in the Lip condition had a total power value that exceeded 3 standard deviations from the mean. The model presented above omits this data point. However, when this participant was included in the analysis, the outcomes (i.e., significance of model terms) did not change. To aid in the visualization of the data and specification of the model estimates, we opted to omit this participant. Inter-trial phase coherence for each stimulus is plotted in Fig. 3C. An increase in 5 to 15 Hz coherence reaches a maximum of 130 to 230 ms across right occipital sensors. Mixed modeling in Table 8 did not reveal any main effects or interactions with stimulus type, high-frequency PTA, or age (*p*’s > 0.15; Fig. 3D).

**Table 7:** 5–15 Hz power

Predictor	Slope	S.E	*F*-statistic	D.F	*p*-value
High-freq. PTA	0.011507	0.006010	3.6661	1, 63.727	0.06002
Condition	0.470768	0.179761	6.8584	1, 62.863	**0.01104**
Age	0.010689	0.018029	0.3515	1, 62.866	0.55538
High-freq. PTA*condition	− 0.011060	0.004429	6.2368	1, 62.643	**0.01515**

*PTA* pure tone average, *S.E.* standard error, *D.F.* degrees of freedom

**Table 8:** 5–15 inter-trial phase coherence

Predictor	Slope	S.E	*F*-statistic	D.F	*p*-value
High-freq. PTA	− 0.0001595	0.0005498	0.0842	1, 65.150	0.77265
Condition	0.0293191	0.0203009	2.0858	1, 63.029	0.15363
Age	− 0.0023203	0.0016323	2.0206	1, 63.975	0.16003
High-freq. PTA*condition	− 0.0001289	0.0005005	0.0663	1, 62.860	0.79767

*PTA* pure tone average, *S.E.* standard error, *D.F.* degrees of freedom

### Source localization of the P2 response

**Fig. 4:**
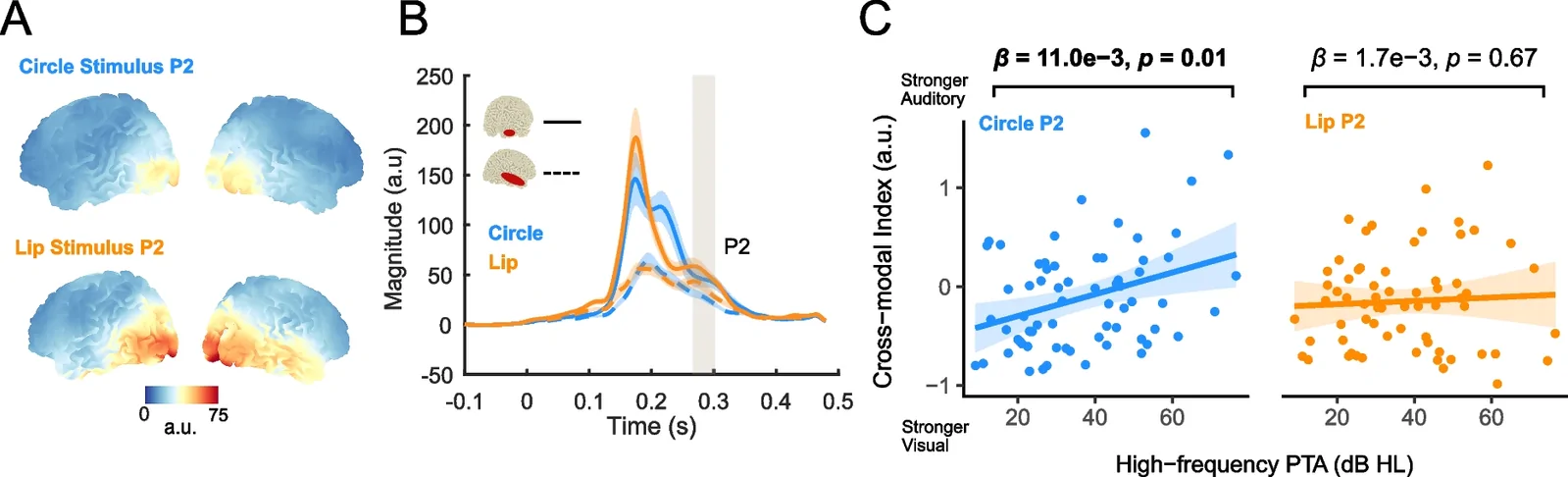
Source estimation of visual P2 response using minimum norm estimation (MNE). **A** Whole-brain plots show cortical P2 activation; each row shows the left and right posterior views, and warmer colors indicate larger activation magnitudes. **B** Source waveforms for auditory (dashed line) and visual (solid line) regions of interest. Solid lines reflect activity from the right inferior occipital cortex, and dashed lines represent the right middle temporal gyrus. ROI locations are shown in the inset legend. Orange and blue reflect Lip and Circle stimuli, respectively. The P2 region is shown as a shaded vertical bar. **C** Scatter plots show the cross-modal activation index, a normalized difference between auditory and visual cortical activation, as a function of high-frequency PTA. Higher values on the ordinate reflect higher cross-modal activation. Solid lines and shaded regions represent mixed model predictions and 95% confidence intervals. Slope estimates and associated *p*-values are shown above each scatterplot.

Source analysis focused on the P2 response since this component showed effects consistent with visual compensatory neuroplasticity. Source activations across the cortex are plotted in Fig. 4A, showing stronger occipital cortex activation and some activation of temporal areas. Occipital activations were strongest in inferior regions, particularly on the right side, while for the temporal lobe, activation was strongest in the middle temporal gyrus. For the right inferior occipital ROI and right middle temporal gyrus shown in Fig. 4B, we extracted activation magnitudes at each individual’s cortical P2 latency and transformed them into the cross-modal activation index (described in the Methods section).

Mixed modeling on the cross-modal activation index (Table 9) returned only a main effect of high-frequency PTA (*p* = 0.028). No other main effects and no interactions were found. The main effect of high-frequency PTA suggests that more hearing loss was associated with greater activation of auditory cortical areas, consistent with cross-modal plasticity. Although we did not detect a difference in activation based on stimulus type, simple slopes analysis in Fig. 4C showed that the effect was qualitatively stronger for the Circle stimulus (*β* = 11.0e − 3, SE = 3.8e − 3, *p* = 0.006) and not for the Lip stimulus (*β* = 1.7e − 3, SE = 3.9e − 3, *p* = 0.67). We questioned whether stronger auditory cortical activation for the Circle stimulus was related to the decline in P2 amplitude at the sensor level. If so, sensor P2 amplitude decline could result from a cross-modal redistribution of visual processing from occipital to temporal cortex [22]. In agreement, higher values of the cross-modal index were associated with lower P2 amplitudes in the Circle stimulus (*r* =  − 0.42, *p* < 0.001) but not the Lip stimulus (*r* =  − 0.11, *p* = 0.41).

**Table 9:** P2 source activation magnitude

Predictor	Slope	S.E	*F*-statistic	D.F	*p*-value
High-freq. PTA	0.006355	0.002830	5.0428	1, 62.039	**0.02830**
Condition	0.305487	0.219679	1.9338	1, 64.095	0.16915
Age	0.001753	0.008384	0.0437	1, 60.320	0.83505
High-freq. PTA*condition	− 0.009304	0.005430	2.9361	1, 63.582	0.09149

*PTA* pure tone average, *S.E.* standard error, *D.F.* degrees of freedom

## Discussion

### Summary

We tested for evidence of compensatory visual plasticity in age-related hearing loss by recording the EEG of 66 middle-aged and older adults as they viewed speech-like and non-speech stimuli. We predicted that cortical VEPs would have shorter latencies and higher amplitudes with worse hearing. Consistent with predictions, visual P2 latencies decreased with increasing hearing loss. Contrary to predictions, the N1 latency increased with more hearing loss selectively for the Lip stimulus. A similar effect was found for the P1 potential, but this effect did not reach significance. P2 amplitudes at the EEG sensor level also decreased with more hearing loss, which for the Circle stimulus further correlated with a shift from intra- to cross-modal activation in source analysis. The Circle stimulus also elicited higher 5–15 Hz power in occipital sensors in participants with worse hearing, a potentially separate compensatory effect. Overall, our findings suggest that the cortical P2 response may reflect visual compensatory plasticity in age-related hearing loss, but N1 and potentially P1 responses for speech-like stimuli may be sensitive to auditory decline.

### Cross-modal and intra-modal plasticity in age-related hearing loss

A shorter P2 latency with hearing loss is consistent with visual compensatory plasticity and was found as a main effect across stimulus types. In contrast, numerical age was associated with longer P2 latencies, revealing diverging effects of hearing loss and age on the visual P2 component. P2 amplitudes also decreased with more hearing loss, which did not agree with our predictions based on past research [14, 22]. However, for the Circle stimulus, the P2 amplitude decrease at the sensor level was associated with increased temporal cortex activation in source analysis, suggesting cross-modal recruitment of auditory areas during motion processing. Therefore, visual compensatory plasticity may be more apparent for later cortical potentials such as the P2, but effects may arise differently depending on stimulus features (i.e., speech versus non-speech motion). We suggest that the visual P2 response is a candidate neural marker for tracking visual compensatory plasticity in age-related hearing loss and other populations. Several lines of research support this conclusion.

Cross-modal plasticity is believed to occur in higher-order, non-primary regions of the sensory cortex and develops through pre-existing top-down connections that upregulate in an experience-dependent manner when neurons in these regions receive reduced input, such as from hearing loss [38]. For this reason, cross-modal effects may be more apparent for later cortical responses such as the P2. The visual P2 response has cortical generators in such higher-order multisensory areas across the parietal, occipital, and temporal cortex [28], and shows plasticity following visual experience and learning [1, 27] including in populations with deafness [46]. The P2 is also sensitive to a variety of stimulus features and cognitive tasks [24, 40, 53] The alignment between the proposed mechanisms of cross-modal plasticity in higher-order areas [38] and the determinants of the P2 response supports the conclusion that this component can track the development of cross-modal plasticity.

Previous research shows that adults with partial hearing loss have shorter P2 latencies elicited by the same Circle stimulus used in this study [13, 30]. Visual compensatory plasticity may account for shorter latencies because it reflects efficiency in sensory regions that respond to visual events [22]. However, the effects of partial hearing loss on the P2 latency are not spatially consistent across the scalp in EEG studies of cross-modal plasticity in mild-to-moderate hearing loss, with some studies showing effects in right temporal but not occipital sensors [30] or in frontal scalp regions [13]. Here, the effects of hearing thresholds on VEP attributes were in parietal and occipital regions.

The discrepancy in P2 effects across the scalp may be attributable to differences in the recruitment or activation of multiple generators that shape the P2 response at the scalp level [28], which could include increased cross-modal recruitment of the temporal cortex. In agreement with this notion, Doucet et al. [22] used the Circle stimulus to study compensatory plasticity in cochlear implant users. Implanted participants with worse speech function had smaller P2 responses in occipital sensors compared to participants with better speech function, matching the findings of this study, but the P2 response distribution shifted to more anterior regions, presumably because of the recruitment of the temporal cortex due to cross-modal plasticity. In contrast, implanted participants with better speech function in the Doucet et al. study had higher P2 amplitudes, more so than typical-hearing controls, which was explained as increased intra-modal plasticity. Therefore, with worse hearing, P2 responses may redistribute from occipital–parietal regions to temporal regions as a cross-modal recruitment effect but visual experience may concurrently drive intra-modal plasticity to increase P2 strength and potentially reduce P2 latencies. P2 amplitude effects are also not consistent in the literature for cases of partial hearing loss, increasing in one study [14] and decreasing in another [13], but effects were measured in different scalp locations and may be tracking different generators of the P2 response. Overall, the present results point to the visual P2 as a viable neural marker of compensatory plasticity in hearing loss that is potentially distinct from the effects of age, although further research is required to clarify how contributions from different neural generators affect P2 morphology. In support of this conclusion, Glick and Sharma [30] found that 6 months of hearing aid use increased VEP latencies including the P2, indicating that visual compensatory effects could be reversed after hearing restoration.

A novel finding in the current study was the increased power of theta/alpha (5–15 Hz) oscillations in participants with worse high-frequency thresholds that, like the P2 cross-modal recruitment effect, was specific to the non-speech Circle stimulus. Past research on visual neural oscillations in hearing loss is limited, but some studies show a reduction or attenuation of oscillation power in  cochlear implant users [60] or individuals with deafness [8], while others show stronger synchronization [55, 56]. Modulations of visual cortical oscillations may therefore be expected with auditory decline, but further research is required to understand their functional significance.

### Visual compensatory plasticity in speech and non-speech visual pathways

The Circle stimulus is routinely used in cross-modal plasticity research and is designed to produce the perception of apparent motion [14, 22, 23, 29, 30], which is relevant because visual cross-modal plasticity causally mediates behavioral motion perception [47]. In contrast, we used the Lip stimulus to determine if visual compensatory plasticity has different effects for speech processing pathways, as has been suggested for cochlear implant users [29]. The Lip stimulus was constructed to permit close comparison to the Circle stimulus, matching features such as the number of frames required to elicit the perception, the inter-onset interval of frames, and eccentricity. Further, the viseme models of the Lip frames were designed to convey audiovisual speech in animation [32, 51]. Importantly, visual speech is known to activate the auditory cortex [12] and has been proposed to be processed in part by the auditory pathway [9], reliant on redundant information shared between sensory channels [15]. If cooperation between audio and visual pathways is required for audiovisual speech perception, visual upregulation in these networks following hearing loss may have different effects than non-speech stimuli.

Differences between speech and non-speech visual processing pathways potentially explain stimulus-specific effects we detected in this study. Statistical interactions between hearing loss and stimulus type were found for P1 and N1 latencies, where longer latencies occurred with more hearing loss for the Lip stimulus (the P1 effect did not reach statistical significance). This disagreed with our predictions for shorter latencies based on past research using the Circle stimulus [13, 14, 30]. We did not find such effects on the Circle stimulus here. Rather, increased latencies for the N1 were specific to the speech stimulus. It is possible that the decline of hearing acuity increased latencies of visual speech N1 (or P1) because visual speech processing relies in part on auditory pathways [9]. Hearing loss is associated with the decline of auditory processing pathways [57] and may similarly affect the processing of visual speech. We speculate that such an effect would be more pronounced for the N1 (and possibly P1) response, if these components are more sensitive to bottom-up influence such as the decline of hearing acuity.

The later cortical P2 did not appear to show a latency increase associated with hearing loss as was found for the P1 and N1 responses, because this stage of the visual processing hierarchy is sensitive to top-down influences and reflects distributed activation from multiple cortical sources [28]. Visual compensation would be expected to occur in these regions because it involves multi-sensory, higher-order areas involved in the processing of stimuli that can be represented by both auditory and visual modalities [38]. In contrast, the visual P1 and N1 responses, when evoked by basic stimuli like checkerboards, are associated with activation in dorsal and ventral extrastriate regions [21]. Extrastriate areas receive modulatory inputs from non-visual sources such as the auditory system [62], underscoring the possibility that the decline of auditory networks influences the processing of visual stimuli if they involve shared activation of auditory regions [12]. However, precise anatomical localization of visual speech-evoked potentials has not been published to our knowledge, and our interpretation is preliminary until this assumption is tested.

A limitation of our view is that we did not include a behavioral task such as lipreading or audiovisual perception. It is not possible to infer if hearing loss-related changes to VEPs for the Lip stimulus reflect behavioral changes to lipreading ability, which would substantiate our claims that changes to the VEP P1 and N1 relate to the hearing loss-related decline of visual speech processing pathways. For future research, we hypothesize that enhanced audiovisual speech integration is associated with modified visual P2 responses, but this effect may be diminished depending on the degree of modulation of P1 and N1 responses by hearing loss. There may be alternative reasons that the VEP P1 and N1 increased in latency with higher levels of hearing loss, such as “common cause” global sensory decline that is not specific to visual speech [2]. However, since we did not observe similar effects for the Circle stimulus, this interpretation is less likely. Our tentative conclusion is that visual neural compensation for the perception of apparent motion possibly involves cross-modal recruitment of the temporal cortex, but compensation for visual speech-like stimuli could implicate intra-modal plasticity in existing (audio)visual speech networks.

### Limitations

This study has limitations. First, EEG source analysis only estimates the location of neural generators, and the spatial resolution of EEG is limited. We did not have magnetic resonance scans or digitized electrode positions from our participants to build individualized head models, and therefore our source analysis used “standard” templates that may have low precision. Source analysis results and ensuing claims about cross-modal recruitment of the temporal cortex should be interpreted cautiously. Second, our conclusions generalize to older adults < 80 years with mild-to-moderate hearing loss. Severer hearing loss would potentially show different effects than those reported here. Third, we did not measure or assess cognitive function in our participants. Previous studies show an association between cognitive function and visual evoked potentials in participants with age-related hearing loss [30]. Given that our participants were older, cognitive factors may also affect VEP amplitude and latency, as well as time–frequency measures. Future studies in older adults on visual cross-modal plasticity should consider accounting for cognitive ability or potential cognitive impairment. Finally, despite feature matching between Lip and Circle stimuli in the degree of visual angle and time dynamics, the image frames differed in their distribution of spatial frequencies and luminance. It is possible that these image differences explain stimulus-specific effects, rather than the degree of similarity of the image to visual speech. In future work, a speech-based stimulus matched in luminance, contrast, spatial frequency, etc., to the non-speech stimulus would better support claims for stimulus selectivity in cross-modal plasticity.

### Conclusions

Our findings suggest that the earlier visual cortical P1 and N1 responses elicited by visual speech may be susceptible to the effects of age-related sensory loss in speech perception networks. This finding opposes claims that P1 and N1 amplitudes and latencies are modulated by compensatory plasticity in individuals with mild-to-moderate cases of hearing loss, as was shown in past research in younger adults exposed to the Circle stimulus [13, 14]. However, the later cortical P2 latency and amplitude may index cross-modal plasticity across the sensory cortex in older adults, unlike the earlier cortical potentials. Cortical generators of the P2 response overlap with higher-order, multi-sensory cortical regions, through which cross-modal plasticity is theorized to develop. We also show evidence that cross-modal effects differ between stimulus types (speech and non-speech stimuli). Although participant age was correlated to hearing sensitivity, it did not confound our analyses. Rather, the effect of age diverged from hearing sensitivity on the P2 response, suggesting that sensory compensation and changes in brain function with age are separate processes. Collectively, these findings show for the first time how sensory compensation interacts with age-associated changes to brain function.

The low cost and ease of use of EEG make it attractive for the development of objective markers of brain plasticity in applied contexts, such as in clinical care. Research in cochlear implant patients suggests that effective use of visual speech has long-term benefits for speech recovery [3, 56, 75]. For older adults with milder hearing loss, the presence of visual speech during auditory speech listening is known to reduce listening effort [61]. Further, older adults’ speech perception may improve with training on lipreading ability, as has been suggested in recent studies in typical populations [66]. In these cases, the VEP P2 response could be used to track visual compensatory neuroplasticity, which could help to guide or select among speech rehabilitation strategies.

## Supplementary Information

Below is the link to the electronic supplementary material.

ESM 1 (PDF 209 KB)

## Data Availability

Data used in statistical analysis will be shared on the Open Science Framework upon publication.
