# Supplementary material for: Visual cortical responses in age-related hearing loss show evidence for compensatory neuroplasticity
Source: GeroScience. 2025 Nov 29;48(5):6739–56. doi: 10.1007/s11357-025-02013-w (PMC13601438; doi:10.1007/s11357-025-02013-w)
Supplement: Supplementary file 1 [file 11357_2025_2013_MOESM1_ESM.pdf]

SUPPLEMENTAL INFORMATION

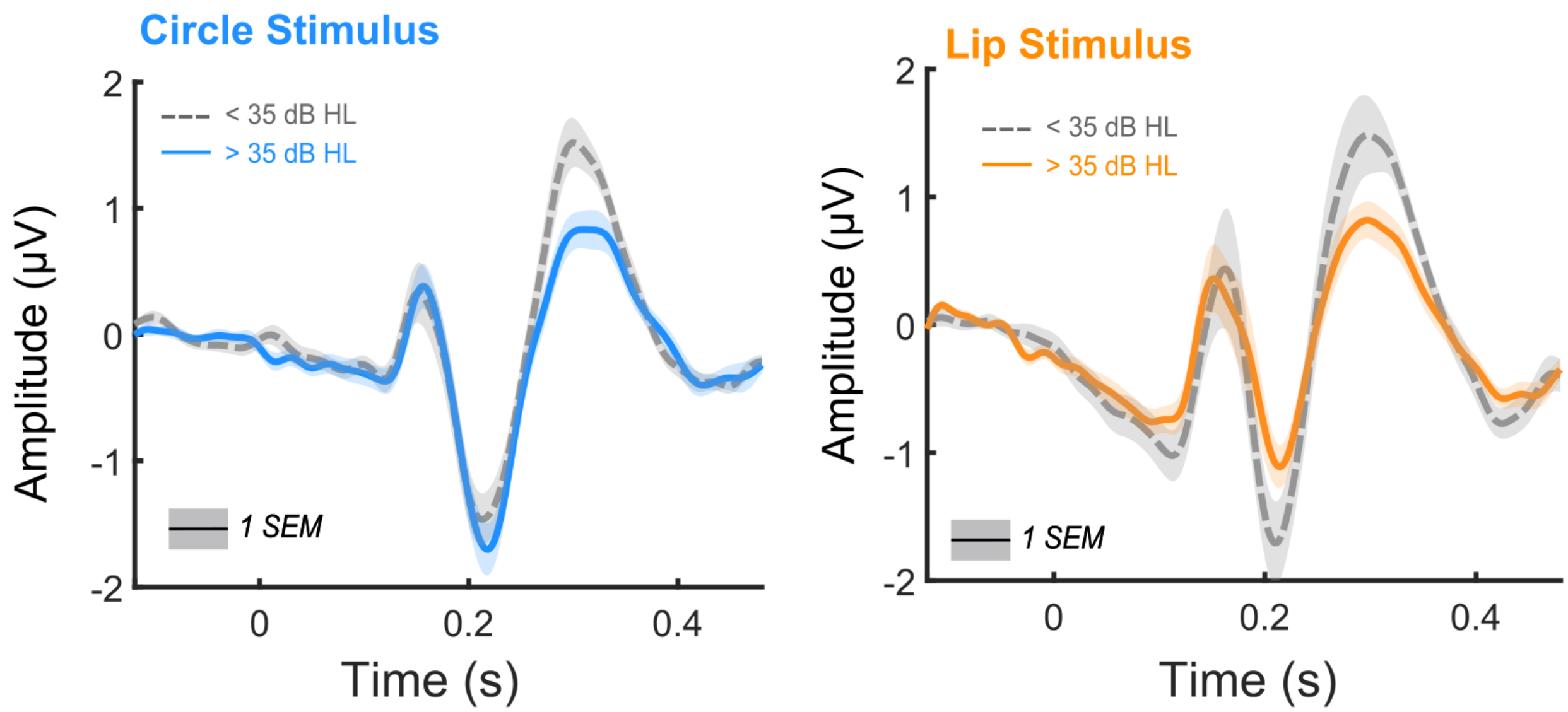

Supplemental Figure 1. Visual evoked potentials for the circle and lip stimuli, for groups of participants with hearing thresholds above 35 dB HL (continuous line) and below 35 dB HL (dashed line). Shaded regions are 1 standard error of the mean.
